# Winogradsky Bioelectrochemical System as a Novel Strategy to Enrich Electrochemically Active Microorganisms from Arsenic-Rich Sediments

**DOI:** 10.3390/mi13111953

**Published:** 2022-11-11

**Authors:** Angela Cantillo-González, Javiera Anguita, Claudia Rojas, Ignacio T. Vargas

**Affiliations:** 1 Departmento de Ingeniería Hidráulica y Ambiental, Pontificia Universidad Católica de Chile, Santiago 7820436, Chile; 2 Centro de Desarrollo Urbano Sustentable (CEDEUS), Santiago 6640064, Chile; 3 Laboratory of Soil Microbial Ecology and Biogeochemistry (LEMiBiS), Institute of Agri-Food, Animal and Environmental Sciences (ICA3), Universidad de O’Higgins, San Fernando 3070000, Chile; 4 Center of Applied Ecology and Sustainability (CAPES), Santiago 8331150, Chile

**Keywords:** sediment microbial fuel cell, biogeochemistry, arsenic, biocathodes, electrochemically active microorganisms

## Abstract

Bioelectrochemical systems (BESs) have been extensively studied for treatment and remediation. However, BESs have the potential to be used for the enrichment of microorganisms that could replace their natural electron donor or acceptor for an electrode. In this study, Winogradsky BES columns with As-rich sediments extracted from an Andean watershed were used as a strategy to enrich lithotrophic electrochemically active microorganisms (EAMs) on electrodes (i.e., cathodes). After 15 months, Winogradsky BESs registered power densities up to 650 μWcm^−2^. Scanning electron microscopy and linear sweep voltammetry confirmed microbial growth and electrochemical activity on cathodes. Pyrosequencing evidenced differences in bacterial composition between sediments from the field and cathodic biofilms. Six EAMs from genera *Herbaspirillum*, *Ancylobacter*, *Rhodococcus*, *Methylobacterium*, *Sphingomonas*, and *Pseudomonas* were isolated from cathodes using a lithoautotrophic As oxidizers culture medium. These results suggest that the tested Winogradsky BES columns result in an enrichment of electrochemically active As-oxidizing microorganisms. A bioelectrochemical boost of centenarian enrichment approaches, such as the Winogradsky column, represents a promising strategy for prospecting new EAMs linked with the biogeochemical cycles of different metals and metalloids.

## 1. Introduction

During the last two decades, the study of bioelectrochemical systems (BESs) has been dedicated to developing technologies for treating organic and inorganic pollutants from water, sediments, and soil. This focus has been based on their environmentally friendly characteristics, cost-effectiveness, versatility, and the potential to recover electrical energy from contaminants [1]. However, BESs could be a promising strategy to enrich and cultivate microorganisms that can replace their natural electron donor or acceptor for an electrode [2]. Usually, these electrochemically active microorganisms (EAMs) are found in metal-rich environments such as soil and sediments, where they must use insoluble electron donors/acceptors to survive [3,4]. 

The EAMs are the critical component of a BES. Research on EAMs has focused on the understanding of extracellular electron transfer (EET) mechanisms used by exoelectrogenic model microorganisms (i.e., *Geobacter*, *Shewanella*, *Pseudomonas*) that use an electrode as a terminal electron acceptor (i.e., anode). In recent decades, increasing attention has concentrated on lithotrophic EAMs capable of uptake and transferring electrons from an electrode (i.e., cathode). Microorganisms from this phenotype are known as electrotrophs [5]. There is not much information on the diversity and mechanisms of electrotrophic microorganisms compared to exoelectrogenic microorganisms [6,7]. For example, microorganisms observed in biocathodic communities, such as *Rhodococcus* sp., *Sphingomonas* sp., or *Herbaspirillum* sp., have been demonstrated to be able to transform and/or resist perchlorate [8], iron (Fe) [9], and arsenic (As) [10], respectively, but not necessarily capable of being isolated or tested in pure culture studies. 

Electrotrophs can be found in diverse environments, and there is a progressive interest in their study due to the enormous potential associated with their use in BESs. However, this type of microorganism is difficult to culture with traditional methods. Electrotrophic microorganisms have been commonly obtained from sediments in mineral-rich environments. Even though the conventional techniques used for culturing and enriching putative electrotrophs are based on defined culture media, there is no consensus about the best growth medium or strategy [11]. For this reason, non-conventional isolation techniques have been developed, such as the U-tube microbial fuel cell [12], the plate-culture electrode [13], and the electrochemical enrichment method used by applying a fixed potential in a three-electrode cell [14,15,16]. 

For decades, the Winogradsky column has been widely used to selectively enrich microorganisms [17,18,19], study microbial diversity [20,21], and understand how microbial communities adapt to polluted sediments and transform them [22]. Recently, Winogradsky columns have been combined with BESs to optimize voltage generation using cellulose as an electron donor [23] and to enrich electroactive communities from an acid mine drainage-affected site [24]. However, to the best of our knowledge, no information on its potential for enriching electrotrophic microorganisms from contaminated environments has been published. 

Andean basins in northern Chile have exhibited active biogeochemical cycles of Fe, sulfur (S), and As [25]. Thus, sediments from these environments are perfect niches for testing enrichment strategies and isolating new microorganisms. The As-oxidizing bacteria *Ancylobacter* TS-1 was recently isolated from a sediment sample extracted from a hydrothermal source in northern Chile [26]. This location presents high As concentrations in sediments (6.4 ± 1.7 mg kg^−1^) and water (0.8 ± 0.2 mg L^−1^), with relatively high electrical conductivity (EC = 3.8 ± 0.1 mS cm^−1^), moderately acidic conditions (pH 5.9 ± 0.1), moderated dissolved oxygen (2.0 ± 0.5 mg L^−1^), and mesophilic temperatures (34.9 ± 0.5 °C) [25]. Bioelectrochemical studies of TS-1 probed its electrotrophic capacity, using an electrode (i.e., cathode) as the only electron donor [27], confirming the potential of this naturally pressured environment as a source for prospecting new EAMs. 

This work combined biogeochemical techniques, molecular tools, scanning electron microscopy, and electrochemistry to evaluate the use of a Winogradsky BES column with As-rich sediments as a direct enrichment strategy for electrochemically active As-oxidizing microorganisms.

## 2. Materials and Methods

### 2.1. Site Description and Sample Collection

**Figure 1:**
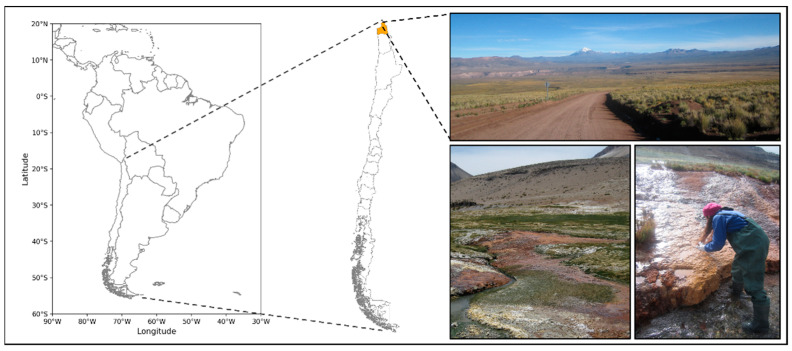
Sampling site located in the upper section of Lluta River (Arica and Parinacota Region) in northern Chile (17°43′12″ S and 69°49′18″ W).

Sediment samples were obtained from a hydrothermal source located in the upper Lluta River (Arica and Parinacota Region) in northern Chile (17°43′12″ S and 69°49′18″ W) (Figure 1). In a first field campaign, sediments were collected in 1000 mL HDPE bottles and transported on ice to the laboratory, where they were kept at 4 °C for seven months until system construction. In a second field campaign, sediments were aseptically collected in 50 mL polypropylene tubes (BD Biosciences, Mountain View, CA, USA) and maintained at 4 °C for DNA extraction within a week of sample collection.

### 2.2. Bioelectrochemical System Set-Up, Operation, and Electrochemical Analysis

**Figure 2:**
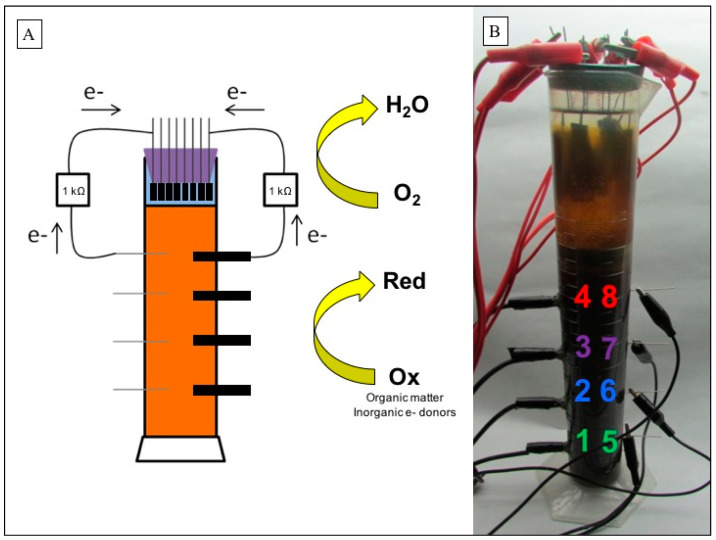
Winogradsky BES system set-up. (**A**) Schematic of the column system constructed with eight carbon felt cathodes (in water), each connected to eight anodes inserted in the sediment. (**B**) Photography of one of the two columns tested in the laboratory. Eight anodes were inserted in the sediment (1–4 graphite rod anodes, 5–8 titanium wire anodes). Colors were used for different cathode–anode distances. red: 7.5 cm, purple: 12 cm, blue: 15.5 cm, and green: 21 cm.

The BESs consisted of duplicated 250 mL graduated cylinders (total volume of 320 mL), each filled with 230 mL of sediments; 40 mL of water from the same hydrothermal source (Table S1), both previously described by Leiva et al. [25]; and 40 mL of deionized filtered water. BESs were configured with eight carbon felt cathodes with a geometric surface of 9.1 cm^2^ (Fuel Cell Store, College Station, TX, USA) submerged in the water zone of the columns at the same depth. Each cathode was connected to an anode through a 1 kΩ resistor. Anodes were made of two different materials, four of graphite (geometric surface of 6.4 cm^2^) and four of titanium (geometric surface of 0.7 cm^2^) (Fuel Cell Store, USA). The anodes of each material were buried in the sediment at different depths (distance between cathode and anode: 7.5 cm, 12 cm, 15.5 cm, and 21 cm) (Figure 2). BESs were operated for 15 months in a temperature-controlled room at ~25 °C, with artificial white light, under closed-circuit conditions. Voltages were measured using a data acquisition system (Multimeter 2700; Keithley, Beaverton, OR, USA). The volume of the columns was maintained over time; for this, deionized filtered water was added when required.

### 2.3. Electrochemical Characterization 

Linear sweep voltammetry (LSV) was used to characterize the electrochemical response of (i) electrodes extracted from the Winogradsky BES and (ii) new electrodes inoculated with bacterial isolates obtained from the Winogradsky BES cathodes. 

Cathodic LSV was performed on electrodes at the end of the BESs operation. Electrodes were removed from the columns and tested using a three-electrode cell. Anodes and cathodes sampled from the columns were independently tested as working electrodes. A counter electrode of platinum (CHI115, CHI Instruments Inc.) and an Ag/AgCl reference electrode (CHI111, CHI Instruments Inc.) were used. The electrolyte consisted of water from the BESs (pH = 4.2 ± 0.1; DO = 8.6 ± 0.1 mg L^−1^; EC = 3.8 ± 0.4 mS cm^−1^). LSVs were performed from 0.1 to −1 V (vs. Ag/AgCl) at 1 mV s^−1^ using a potentiostat Reference 600 (GAMRY, Warminster, PA, USA).

The electrochemical characterization of the isolates was performed using the same three-electrode cell described above, filled with PBS (10 mM), and inoculated with each bacterial isolate (i.e., independent tests for each isolate). The bacterial concentration was adjusted to an optical density of 0.4 at 600 nm. The working electrodes were new sterile pieces of carbon felt (Fuel Cell Store, College Station, TX, USA) with geometric surfaces of 9.1 cm^2^, a counter electrode of platinum (CHI115, CHI Instruments Inc., Bee Cave, TX, USA), and an Ag/AgCl reference electrode (CHI111, CHI Instruments Inc.). LSVs were performed from 0.2 to −1.0 V (vs. Ag/AgCl) at 1 mV/s using a potentiostat Reference 600 (Gamry Instrument Inc.).

### 2.4. Scanning Electron Microscopy (SEM) and Bacterial Isolation from BES Cathodes

A portion of each cathode (0.27 g wet weight; corresponding to ~20% of the electrode) was fixed with 2% glutaraldehyde, dried using a critical point, and coated with silver before microscopic observation. Microbial growth on the surface of the cathode electrodes was confirmed by scanning electron microscopy (SEM). A LEO 1420VP scanning electron microscope coupled to an Oxford 7424 solid-state detector was used for microscopic and energy dispersive spectroscopy (EDS) analyses. 

A second part of each cathode (0.27 g fresh weight) was used to isolate potential As-oxidizing microorganisms. A basal growth medium was prepared using (per liter of water): 1299 mg NaAsO_2_; 8400 mg NaHCO_3_; 30 mg Na_2_SO_4_; 100 mg KCl, 80 mg MgCl_2_; 100 mg CaCl_2_·2H_2_O; 200 mg (NH_4_)_2_SO_4_; 6.8 mg KH_2_PO_4_; 0.018 mg AlCl_3_·6H_2_O; 0.03 mg Na_2_WO_4_·2H_2_O; 0.2 mg Na_2_EDTA, HCl 3.7%, trace elements added according to Bahar [28], and vitamins [29]. Each cathode portion was placed in 5 mL of the final culture media and maintained in a rotatory shaker (at 200 rpm and 30 °C) until turbidity was observed (about four weeks after incubation). After incubation, aliquots (100 µL of liquid culture) were spread homogeneously on Petri dishes with the same media described above but solidified using agar 1.5% (BD Difco&trade Bacto™). When growth was observed, colonies with different morphologies and colors were transferred to new solid media successively to obtain isolates. DNA was extracted from isolates using a Genomic DNA Kit (PureLink^®^), following the manufacturer’s instructions. The 16S rRNA coding genes were amplified using primers 8F (3′-AGAGTTTGATCCTGGCTCAG-′5) and /1392R (3′-ACGGGCGGTGTGTAC-′5). PCR products were purified and sequenced by Macrogen Inc. (Seoul, Korea). Sequences were compared against sequences of type strains using the classifier tool from Ribosomal Database Project (RDP) release 11 [30]. Additionally, to identify the nearest taxa, sequences were subjected to BLASTn. These sequences were aligned with the closest matches found in the GenBank database (http://www-ncbi-nlm-nih-gov.pucdechile.idm.oclc.org/ accessed on 25 August 2022) using the ClustalW tool from MEGA X [31].

The 16S rRNA gene sequences were deposited in the GenBank database under accession numbers OP379285-OP379286-OP379287-OP379288-OP379289-OP379290-OP379291-OP379292-OP379293-OP379294.

### 2.5. Microbial Community Characterization 

Community DNA was extracted from two sediment subsamples (0.25 g fresh weight) obtained from the field (2nd campaign) and from representative cathodes from both BESs (0.27 g fresh weight) at the end of the incubation period using the Power Soil^®^ DNA isolation kit (MoBio Laboratories, Inc., Carlsbad, CA, USA) according to the manufacturer´s protocol. All DNA concentrations were measured using a NanoDrop 2000c spectrophotometer ( Thermo Fisher Scientific, Waltham, MA, USA). Community DNAs were subjected to bar-coded amplicon library preparation by PCR to amplify 16S rRNA genes using the primers 28F (5′-GAGTTTGATCNTGGCTCAG-3′) and 519R (5′-GTNTTACNGCGGCKGCTG-3′). Targeted sequences were then pyrosequenced using the 454 FLX Titanium system at the Research and Testing Laboratory, (Lubbock, TX, USA). Pyrosequencing data were processed and analyzed with the Quantitative Insights Into Microbial Ecology (QIIME) software v.1.9.0 [32].

## 3. Results and Discussion 

### 3.1. Operation and Electrochemical Analysis of Winogradsky BESs

**Figure 3:**
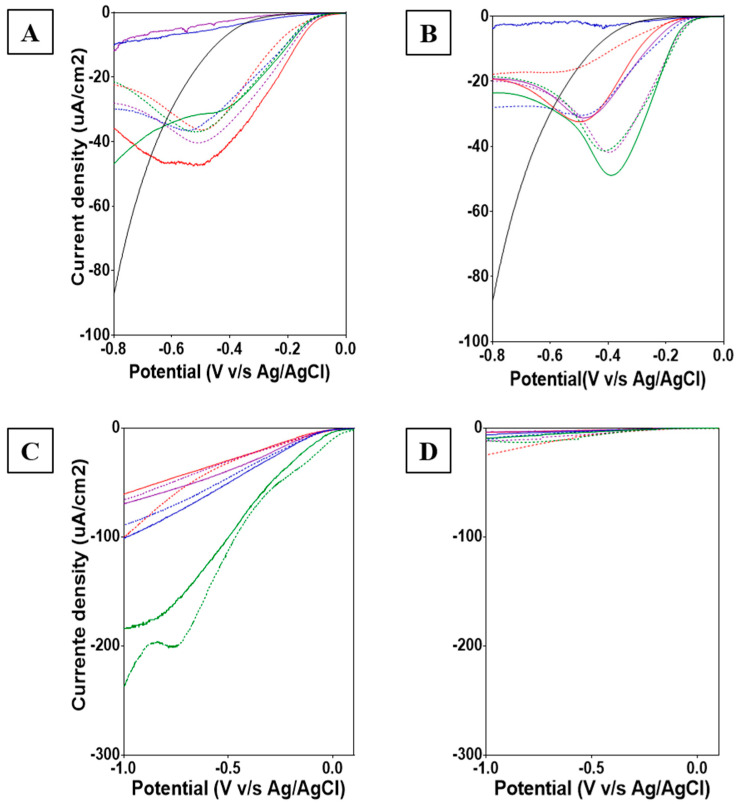
Linear Sweep Voltammetry (LSV) for electrodes from BES after 15 months. (**A**) Cathodes connected to graphite anode. (**B**) Cathodes connected to titanium anodes. (**C**) Graphite anodes. (**D**) Titanium anodes. Colors were used to represent each system with anodes at different depths: red 7.5 cm, purple 12 cm, blue 15.5 cm, and green 21 cm. Continue lines were used for column 1 (BC1) and discontinue lines for column 2 (BC2).

During the 15 months of operation, BESs produced power densities ranging from 650 μWcm^−2^ for the first two months of operation to low power in the range of nano Watt per square centimeter (nWcm^−2^) at the end of the experiment (Figure S1). These values are comparable to similar systems, such as constructed wetlands integrated into microbial fuel cells (MFCs) [33], and marine sediments or soil MFCs [34]. LSVs conducted on biocathodes of the tested columns revealed a shift in the cathodic potential from about −0.2 V (vs. Ag/AgCl) observed in abiotic controls to 0.0 V (vs. Ag/AgCl) (Figure 3A,B). Biocathodes connected to graphite and titanium anodes presented potential peaks ranging from −0.4 to −0.55 V (vs. Ag/AgCl). 

LSV tests conducted for Winogradsky anodes revealed differences between graphite and titanium electrodes. While LSVs for graphite electrodes suggest the development of electrochemically active biofilms, titanium electrodes do not show current. The affinity of EAMs for carbon-based instead of metallic electrodes could allow the separation of microbial from galvanic potential in a sediment column (Figure 3C,D). Additionally, LSVs conducted on graphite anodes show an effect caused by the depth of electrode insertion in stratified sediments on biocathode performance. This result suggests that the cathodic biofilm formed on electrodes at level 1 (green lines) could be able to reduce an inorganic compound (e.g., sulfate, arsenic, iron) present in the sediment. Although graphite anodes showed catalytic activity, they did not present clear peaks; thus, electrochemical and microbial community characterization was focused on the formed biocathodes.

Cathodes associated with graphite anodes inserted in the deeper part of the sediment of each column (green lines in Figure 3) were selected to compare the different electrochemical performances observed among replicates (Figure 3A,B). Biocathodes from column 1 (BC1) showed a cathodic current peak of 30 μA cm^−2^ at −0.4 V vs. Ag/AgCl. This peak has been previously linked to *Acidithibacilus ferrooxidans* catalyzing oxygen reduction [2]. In contrast, biocathodes from column 2 (BC2) showed a clear cathodic current plateau of 37 μA cm^−2^ at −0.5 V vs. Ag/AgCl, revealing a different catalytic effect. A cathodic peak at −0.5 V (vs. Ag/AgCl; pH = 2.6) was previously observed in carbon brush biocathodes dominated by *Acidithiobacillus* of similar Winogradsky BES columns with acid mine drainage sediments [24]. This bacterium has been reported as an Fe(III) reducer [35,36]. 

Differences observed in cathodic potential could be associated with different microbial communities with different EAMs catalyzing electron transfer from electrodes to the medium.

### 3.2. Bacterial Community Characterization 

**Figure 4:**
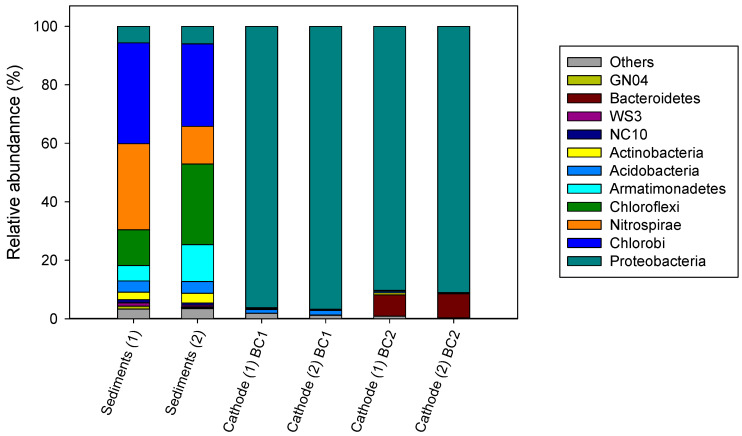
Relative abundances of bacteria phyla in sediments and cathodes from columns BC1 and BC2 (21 cm of cathode–anode distance, green line in Figure 3). The number in parentheses represents the replicate. The bacterial group *others* consisted of sequences of other phyla comprising less than 0.1% of classified sequences.

A total of 28 phyla were detected in sediment samples used to construct the Winogradsky BES columns. The sediment bacterial communities were dominated mainly by the phyla Chlorobi, Chloroflexi, and Nitrospirae (Figure 4). These phyla have been reported as groups present in As-rich environments in the same region of Chile [37,38,39] as in other arsenic-rich environments, such as rice soil in India [40] and China [41,42]. Moreover, previous studies have demonstrated that genes related to As redox reactions are present in diverse phylogenetic groups of prokaryotes, including members of Proteobacteria, Chlorobi, Chloroflexi, and Nitrospirae [43,44]. 

Selection and enrichment may occur within the columns and the biofilms developed on the inserted electrodes. Only 15 phyla were detected from enriched biocathodes. In both cathodic biofilms, Proteobacteria was the most abundant phylum (BC1 ~90% and BC2 ~96%). The Xanthomonadaceae family was the most dominant Proteobacteria in BC1 (~46%), whereas Acetobacteraceae was in BC2 (92%). Recently, the Xanthomonadaceae family was identified as an important member (6%) of an electroactive denitrifying biofilm in biocathodes of BESs [45], and the presence of Acetobacteraceae has also been reported in denitrifying cathodes [46]. Additionally, members of this taxa have shown Fe oxidation capability [47]. Acetobacteraceae was dominated mainly by *Acidocella* species in both biocathodes (BC1 ~16% and BC2 ~27%). *Acidocella* species are known dissimilatory Fe(III) reducers that play a role in Fe(III) oxy(hydr)oxides dissolution [48]. As these microorganisms can indirectly facilitate changes in As mobilization in the environment, their abundance in cathodic biofilms demands further investigation. 

### 3.3. SEM-EDS Characterization of Biocathodes

**Figure 5:**
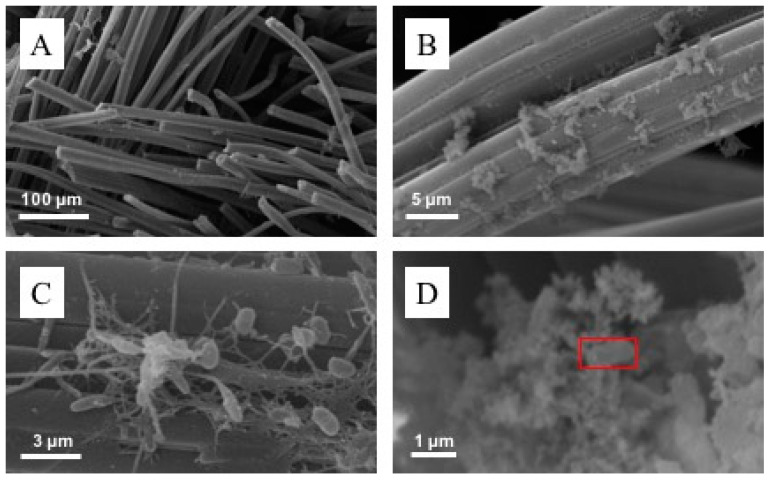
Scanning electron micrographs of carbon felt biocathodes. (**A**) Electrode fibers (magnification 500X. (**B**) Early biofilm formation over fibers (magnification 5000X). (**C**) Microorganisms and extracellular polymeric substances-like structures formed over electrodes (magnification 5000X). (**D**) Mineral precipitates formed over the biofilm. The red square represents the surface area analyzed by EDS.

Microbial colonization of cathodes was confirmed by SEM. Images evidenced rod-shaped microorganisms, and early biofilm formation on the carbon felt fibers (Figure 5). Figure 5A shows the morphology of the carbon felt fibers of the cathodes. Figure 5B,C revealed biofilm formation on the electrodes in cluster-like arrangements and microbial cells connected by fiber-like structures. Figure 5D shows mineral precipitates on the surface of the fibers and spatially linked with microorganisms. EDS analysis of minerals formed along with microorganisms (red square in Figure 5D) revealed the presence of Fe and S as part of the by-product structures (Figure S2). This finding suggests the presence of microbial communities involved in the cycling of S and Fe in sediments and water. No As was detected as part of the formed precipitates. 

### 3.4. Identification and Characterization of Isolates from Winogradsky BES Cathodes

**Figure 6:**
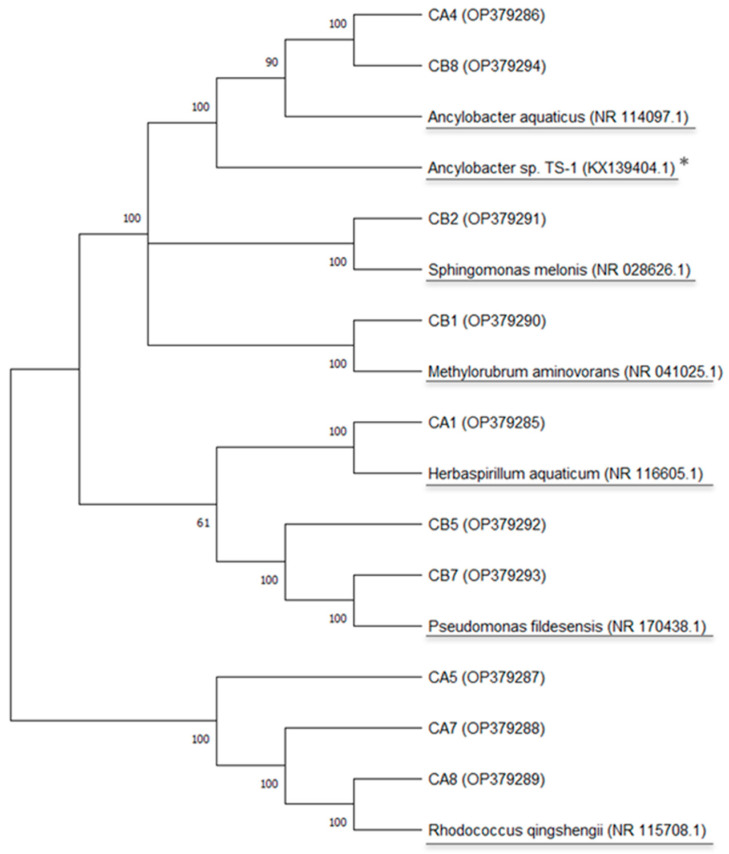
Neighbor-joining phylogenetic tree based on the 16S rRNA sequences of the isolates obtained from cathodes. Reference sequences retrieved from GenBank (underlined) were added for comparison. Genbank number in parentheses. Bootstrap values (1000 replicates) are shown at the tree nodes. (*) The microorganism *Ancylobacter* TS-1 was previously isolated from the same site where sediments were collected.

Bacteria were isolated from cathodes using a culture medium for lithoautotrophic As oxidizers, which suggests the As resistance of isolates and their role in its chemistry. The 16S rRNA gene sequence analysis (~1100 bp) showed that isolates belonged to the Actinobacteria and Proteobacteria phyla. A total of six microorganisms were isolated from BC1 and BC2 (Table 1). The first obtained isolate (i.e., CA1) had a 100% confidence threshold close to *Herbaspirillum*. This microorganism has been found in rhizosphere soil [49] and groundwater with high As concentrations [10]. In addition, reports have shown its resistance to metals such as copper, zinc, lead, and metalloids such as arsenic [50,51]. The As resistance in this microorganism has been correlated with low molecular weight protein tyrosine phosphatases [52]. The second isolate (i.e., CA4 and CB8) presented a 97% confidence threshold close to the *Ancylobacter*. *Ancylobacter* sp. TS-1 was isolated from the same site where the sediments were collected. Interestingly, chemolithoautotrophic As oxidation was probed for TS-1 [26], along with its capacity to form biofilms [53], and the use of the cathode as the only electron donor [27]. The third obtained isolate (i.e., CA5, CA7, CA8) was close to *Rhodococcus* with a 100% identity confidence threshold. While bacteria belonging to the genus *Rhodococcus* have not been reported as electrochemically active, they have been associated with the transformation of many contaminants such as polycyclic aromatic hydrocarbons, nitriles, and phenolic compounds [54], and as a dominant genus in BES communities [55,56]. The fourth isolate (i.e., CB1) showed a 100% confidence threshold close to *Methylobacterium*. Some species of *Methylobacterium* have been reported to oxidize thiosulfate [57], revealing lithotrophic capabilities. The presence of members of this genus has been reported as part of anodic [58] and cathodic [59] communities. Indeed, *Methylobacterium* extorquens has been used in biocathodes for the biosynthesis of formate [60]. The fifth isolate obtained from the cathodes (i.e., CB1) was close to *Sphingomonas* with a 100% confidence threshold. The electrochemically active *Sphingomonas* DJ strain was isolated from a microbial electrolysis cell designed for wastewater treatment. This microorganism has demonstrated the capacity to transform bromoamine acid [56], azo dye methyl red [61] and Fe(III) reduction [9]. Finally, the sixth isolate obtained (i.e., CB5, CB7) was close to *Pseudomonas* with a 100% confidence threshold. *Pseudomonas* species have been previously reported in BES related to wastewater treatment [62], used as model EAM to test BES architecture [63], and electrochemically evaluated in co-culture studies [64,65]. Indeed, *Pseudomonas aeruginosa* has been syndicated as model EAM using phenazine as external shuttling molecules, which act as an EET mechanism in anaerobic conditions [66,67]. Furthermore, purified phenazine could be used as an electron mediator by other microorganisms improving the electricity generation of a BES [68]. A multiple sequence alignment guide tree (Figure 6) was constructed to show the phylogenetic diversity of the ten microorganisms (six different isolates) obtained from the Winogradsky BES cathodes. Table S2 presents the taxonomy assignment by BLAST.

**Figure 7:**
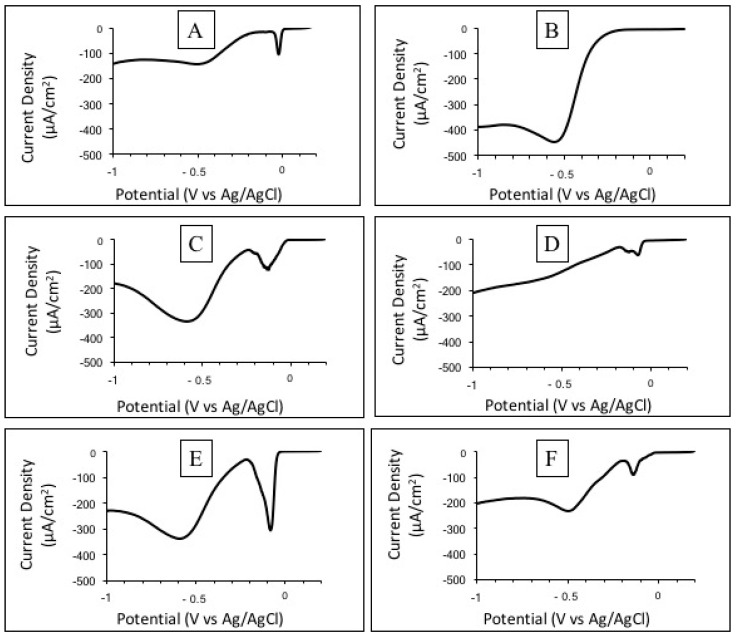
LSV tests conducted on isolates obtained from biocathodes. Representative volumetric profiles of isolates: (**A**) *Herbaspirillum*. (**B**) *Ancylobacter*. (**C**) *Rhodococcus*. (**D**) *Methylobacterium*. (**E**) *Sphingomonas*. (**F**) *Pseudomonas*.

Microbial isolates were electrochemically characterized by LSV tests (Figure 7). Two cathodic peaks were identified. A peak close to −0.1V (vs. Ag/AgCl) was observed for five of the six isolates, excluding the one close to the genus *Ancylobacter* (Figure 7B). This peak has been previously reported by Citrobacter sp. KVM11, isolated from microbial electrochemical remediation systems and associated with Fe(III) reduction [69]. A second peak close to −0.55 V (vs. Ag/AgCl) was observed in all isolates with current densities ranging from −125 to −447 µA cm^−2^. Interestingly, the only isolate obtained from both columns (i.e., BC1 and BC2) was the one belonging to the genus *Ancylobacter*. The LSV results revealed no peak at −0.1 V (vs. Ag/AgCl) and a clear cathodic peak at −0.55 V (vs. Ag/AgCl) with a cathodic current density as high as −413 ± 48 µA cm^−2^, similar to the −0.5 V (vs. Ag/AgCl; pH = 7.2) previously obtained for *Ancylobacter* TS-1, a microorganism directly isolated from the same As-rich sediments used in the columns. The electrotrophic capacity of the As-oxidizing microorganism *Ancylobacter* TS-1 was probed after its isolation in a three-electrode cell [27]. Thus, the molecular and electrochemical characterization of this isolate suggests that TS-1 was enriched in the Winogradsky BES biocathodes. A summary of the cathodic current registered at −0.55 V (vs. Ag/AgCl) is presented in Table S3. 

## 4. Conclusions

This work represents a first step in using a Winogradsky BES as an enrichment strategy for EAMs in sediments rich in As, Fe, and S. The results suggest the effect of BESs as an enrichment of microorganisms of the family Xanthomadaceaea (~46% of column 1), a putative electrotrophic denitrifying bacteria, and (ii) the genus *Acidocella* (16% and 27% of columns 1 and 2, respectively), an Fe-reducer, able to solubilize Fe(III) oxy(hydr)oxide. Dissolution of Fe-rich precipitates could result in the release and mobilization of arsenate into the water. In addition, six electrochemically active bacterial isolates were obtained from biocathodes and successfully grown in a culture medium with As as the only electron donor, suggesting lithotrophic capabilities. Microorganisms were close to genera *Herbaspirillum*, *Ancylobacter*, *Rhodococcus*, *Methylobacterium*, *Sphingomonas*, and *Pseudomonas*. Thus, this work evidences the potential of using a Winogradsky BES as a strategy to enrich and isolate EAMs microorganisms related to As biogeochemistry, with a high potential to be used for treating contaminants in a BES. Further research is required to expand the enrichment opportunities of Winogradsky BES using sediments with a high concentration of other metals, metalloids, or contaminants. 

## Figures and Tables

**Table 1:** Taxonomy of the obtained isolate. Assignment according to the Ribosomal Database Project classifier.

Isolate ID	Closed Bacteria	Confidence Threshold
CA1	*Herbaspirillum* sp.	100%
CA4	*Ancylobacter* sp.	97%
CA5	*Rhodococcus* sp.	100%
CA7	*Rhodococcus* sp.	100%
CA8	*Rhodococcus* sp.	100%
CB1	*Methylorubrum* sp.	100%
CB2	*Sphingomonas* sp.	100%
CB5	*Pseudomonas* sp.	100%
CB7	*Pseudomonas* sp.	100%
CB8	*Ancylobacter* sp.	97%

## Data Availability

All 16S rRNA gene sequences of isolates described in the manuscript are deposited in NCBI GenBank database under accession numbers mentioned in the body of the manuscript.

## Supplementary Materials

The following supporting information can be downloaded at: https://www.mdpi.com/article/10.3390/mi13111953/s1. Figure S1: Power production during operation. Figure S2: Energy dispersive analysis of mineral precipitates observed over cathode electrodes. Table S1: Physicochemical characterization of sediments used for the Winogradsky-BES columns. Table S2: Isolate’s taxonomy assignment by BLAST. Table S3: Current density (µA cm^2^) at −0.55 V (vs. Ag/AgCl) of the isolates.
