# Supplementary material for: Winogradsky Bioelectrochemical System as a Novel Strategy to Enrich Electrochemically Active Microorganisms from Arsenic-Rich Sediments
Source: Micromachines (Basel). 2022 Nov 11;13(11):1953. doi: 10.3390/mi13111953 (PMC9692521; doi:10.3390/mi13111953)
Supplement: Supplementary file 1 [file micromachines-13-01953-s001.zip › micromachines-1954192-supplementary.pdf]

Supplementary Information

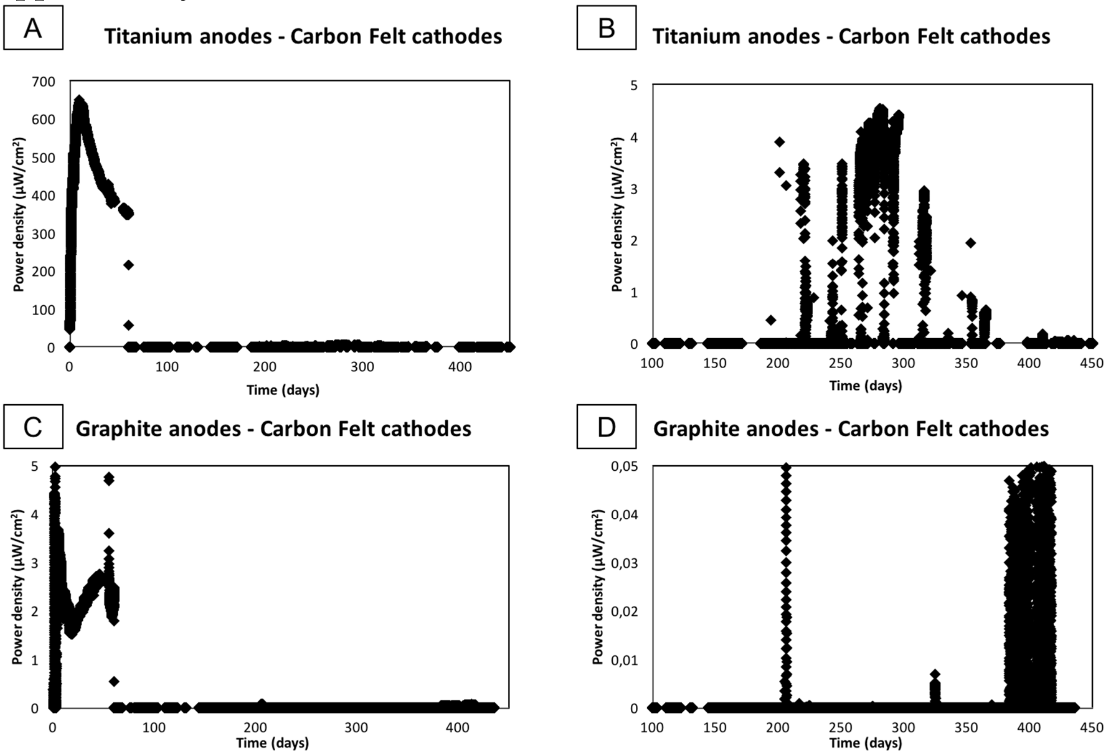

Figure S1. Power production during operation. (A) Average power density registered for titanium anodes – carbon felt cathodes cells during the 450 days of operation. (B) Average power density registered for titanium anodes – carbon felt cathodes cells after the first 100 days of operation. (C) Average power density registered for graphite anodes – carbon felt cathodes cells during the 450 days of operation. (B) Average power density registered for graphite anodes – carbon felt cathodes cells after the first 100 days of operation.

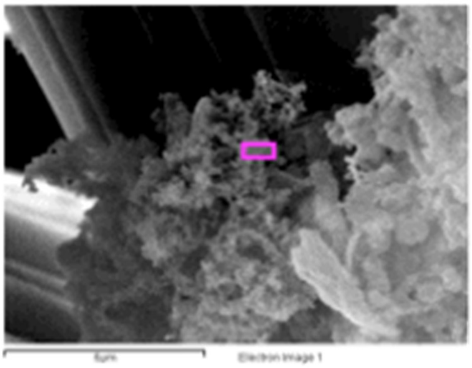

Element	Weight%	Atomic%
C K	88.23	92.06
O K	9.00	7.05
S K	0.96	0.37
Ca K	1.17	0.37
Fe K	0.64	0.14
Totals	100.00	

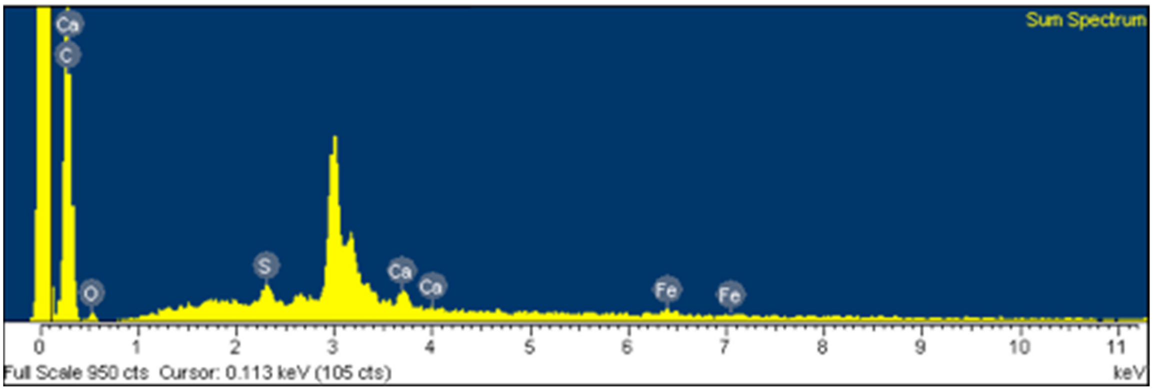

Figure S2. Energy dispersive analysis of mineral precipitates observed over cathode electrodes.

Table S1. Physicochemical characterization of sediments used for the Winogradsky-BES columns. [25]

Parameter.	Value
pH	~ 5.8
Conductivity	~ 4 mS cm^{-1}
Arsenic	~ 6 mg kg^{-1}
Sulfur	~ 6 g Kg^{-1}
Iron	~ 300 g Kg^{-1}

Table S2. Isolate's taxonomy assignment by BLAST.

Isolate ID	Closed bacteria	GenBank number	Query cover (%)	Identity (%)
CA1	*Herbaspirillum aquaticum*	NR_116605.1	100	99,82
CA4	*Ancylobacter aquaticus*	NR_114097.1	100	98,64
CA5	*Rhodococcus qingshengii*	NR_115708.1	100	100
CA7	*Rhodococcus qingshengii*	NR_115708.1	100	100
CA8	*Rhodococcus qingshengii*	NR_115708.1	100	100
CB1	*Methylorubrum aminovorans*	NR_041025.1	100	99,91
CB2	*Sphingomonas melonis*	NR_028626.1	100	100
CB5	*Pseudomonas fildesensis*	NR_170438.1	100	99,83
CB7	*Pseudomonas fildesensis*	NR_170438.1	100	99,91
CB8	*Ancylobacter aquaticus*	NR_114097.1	100	98,64

Table S3. Current density (µA cm^2) at −0.55V (vs. Ag/AgCl) of the isolates.

Isolate	Current density (µA cm^2) at -0.55 V vs. Ag/AgCl
Cathode 1A	−141 ± 37
Cathode 4A	−140 ± 13
Cathode 5A	−175 ± 3
Cathode 7A	−294 ± 27
Cathode 8A	−226 ± 52
Cathode 1B	−125 ± 16
Cathode 2B	−284 ± 129
Cathode 5B	−231 ± 71
Cathode 7B	−447 ± 38
Cathode 8B	−413±91
Abiotic control	−118 ± 24
